# Poly[penta­kis­(μ-cyanido-κ^2^ *N*:*C*)tris­(5-phenyl-2,2′-bipyridine-κ^2^ *N*,*N*′)penta­copper(I)]

**DOI:** 10.1107/S1600536811045910

**Published:** 2011-11-09

**Authors:** Shuxin Cui, Minghui Zuo, Jingping Zhang, Yulong Zhao, Rongxin Tan, Shujuan Liu, Shuangyue Su

**Affiliations:** a College of Chemistry and Chemical Engineering, Mu Danjiang Normal University, Mu Danjiang 157012, People’s Republic of China; b Faculty of Chemistry, Northeast Normal University, Changchun 130024, People’s Republic of China

## Abstract

The hydro­thermal reaction of Cu(acetate)_2_ and K_3_[Fe(CN)_6_] with 5-phenyl-2,2′-bipyridine (5-ph-2,2′-bpy) in water yields the polymeric title complex, [Cu_5_(CN)_5_(C_16_H_12_N_2_)_3_]_*n*_, which consists of ribbons along the *a* axis, constructed from 26-membered {Cu_10_(CN)_8_} rings. In these rings, the metal atoms are bridged by cyanide groups, except for one close Cu⋯Cu contact [2.7535 (12) Å], which can be considered as ligand-unsupported. Within the rings, one Cu atom has a distorted tetra­hedral geometry through the coordination to two N atoms from 5-ph-2,2′-bpy and two N/C atoms from two cyanide groups. Two Cu atoms have a trigonal planar environment being coordinated by three cyanide groups and two other Cu atoms have a distorted square planar geometry through coordination to two N atoms from 5-ph-2,2′-bpy and two N/C atoms from two cyanide groups.

## Related literature

For applications of coordination metal complexes related to the title complex, see: Kong *et al.* (2008 ▶); Ohba *et al.* (2008 ▶). For related complexes containing short unsupported Cu⋯Cu contacts, see: Zhang *et al.* (2005 ▶, 2008 ▶); Chen *et al.* (2009 ▶).
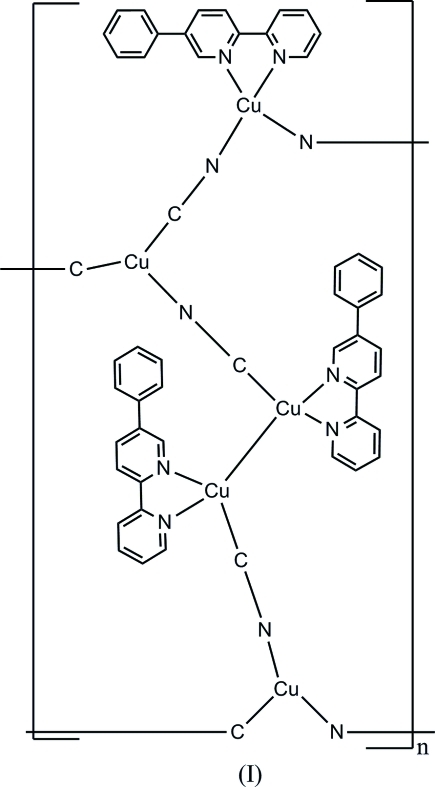


## Experimental

### 

#### Crystal data

[Cu_5_(CN)_5_(C_16_H_12_N_2_)_3_]
*M* *_r_* = 1144.63
Orthorhombic, 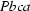

*a* = 32.132 (8) Å
*b* = 8.361 (2) Å
*c* = 34.836 (9) Å
*V* = 9359 (4) Å^3^
*Z* = 8
Mo *K*α radiation
μ = 2.29 mm^−1^
*T* = 153 K
0.39 × 0.09 × 0.05 mm

#### Data collection

Bruker SMART CCD area-detector diffractometer
Absorption correction: multi-scan (*SADABS*; Sheldrick, 2004 ▶) *T*_min_ = 0.777, *T*_max_ = 0.884
50069 measured reflections
9334 independent reflections
5271 reflections with *I* > 2σ(*I*)
*R*_int_ = 0.101

#### Refinement

*R*[*F* ^2^ > 2σ(*F* ^2^)] = 0.063
*wR*(*F* ^2^) = 0.124
*S* = 1.05
9334 reflections
622 parameters
H-atom parameters constrained
Δρ_max_ = 0.57 e Å^−3^
Δρ_min_ = −0.31 e Å^−3^

### 

Data collection: *SMART* (Bruker, 2007 ▶); cell refinement: *SAINT* (Bruker, 2007 ▶); data reduction: *SAINT*; program(s) used to solve structure: *SHELXS97* (Sheldrick, 2008 ▶); program(s) used to refine structure: *SHELXL97* (Sheldrick, 2008 ▶); molecular graphics: *XP* in *SHELXTL* (Sheldrick, 2008 ▶); software used to prepare material for publication: *SHELXL97*.

## Supplementary Material

Crystal structure: contains datablock(s) I, global. DOI: 10.1107/S1600536811045910/vn2020sup1.cif

Structure factors: contains datablock(s) I. DOI: 10.1107/S1600536811045910/vn2020Isup2.hkl

Additional supplementary materials:  crystallographic information; 3D view; checkCIF report

## Figures and Tables

**Table 1:** Selected bond lengths (Å)

Cu1—C16	1.871 (5)
Cu1—N11	1.978 (6)
Cu1—N2	2.095 (5)
Cu1—N1	2.098 (4)
Cu2—C17	1.857 (6)
Cu2—N8	1.924 (4)
Cu2—N7	1.958 (5)
Cu3—C18	1.862 (5)
Cu3—N4	2.029 (4)
Cu3—N3	2.044 (4)
Cu3—Cu4	2.7535 (12)
Cu4—C36	1.859 (6)
Cu4—N6	2.041 (4)
Cu4—N5	2.062 (4)
Cu5—C35	1.874 (6)
Cu5—N9	1.942 (4)
Cu5—N10	1.982 (5)

**Fig. 1.**
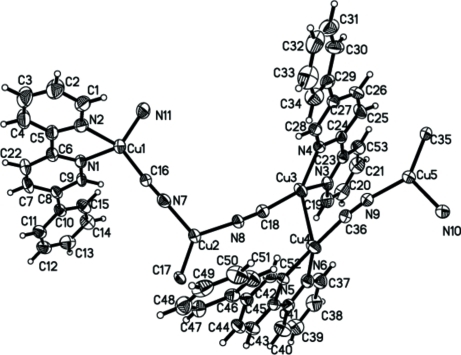
Complex (I), with the atom-numbering scheme. Displacement ellipsoids are drawn at the 50% probability level and H atoms are shown as small spheres of arbitrary radii.

**Fig. 2.**
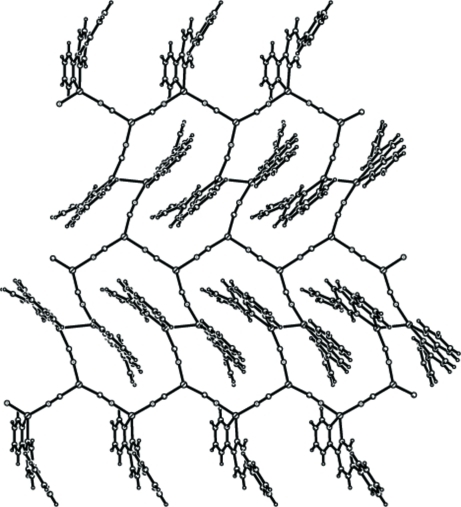
A perspective view of polymer ribbon of complex (I).

**Fig. 3.**
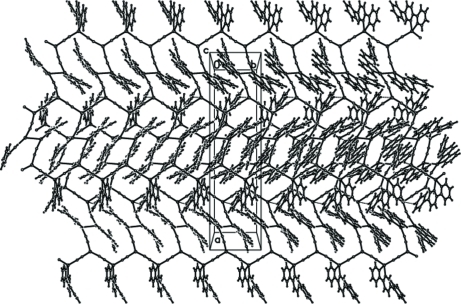
A view along the c axis of the network structure of complex (I).

## supplementary crystallographic information

### Comment

The coordination metal complexes containing cyanide-bridged Cu atoms have been shown to exhibit fascinating structures and interesting magnetic properties (Kong *et al.*, 2008; Ohba *et al.*, 2008). Our studies here aimed at constructing such a copper compound using cyanide and 5-phenyl-2,2'-bipyridine (5-ph-2,2'-bpy) as the ligands. We report here the crystal structure of the title complex (I).

The asymmetric unit of the structure of (I) together with the atomic labeling scheme is given in Figure 1. The structure consists of a one-dimensional ribbon (Figure 2), constructed from {Cu_10_(CN)_8_} rings. The 26-membered {Cu_10_(CN)_8_} rings are defined by the sequence {(Cu5)_3_—Cu4—Cu3—Cu2—Cu1—Cu2—Cu3—Cu4}, in which metal centers except Cu3 and Cu4 centers are bridged by cyanide groups. Each 26-membered ring shares 4 edges with 4 adjacent 26-membered rings to form a unique herringbone pattern (Figure 2). The 26-membered rings have dimensions of *ca* 11.8 * 10.8 Å from vertex to opposing vertex. Within these rings, the Cu1 atom show distorted tetrahedral geometry through coordination to two N atoms from 5-ph-2,2'-bpy as terminal ligand and two N/C atoms from two cyanide groups, bridging Cu1 to two Cu2 (Cu2 and Cu2^i^) [symmetry codes: (i)+*x*, -1+*y*,+*z*] atoms. Cu2 and Cu5 atoms exhibit trigonal planar environment, being coordinated by three cyanide groups, two bridging Cu2 to two Cu1 (Cu1 and Cu1^ii^) [symmetry codes: (ii)+*x*, 1+*y*,+*z*] atoms and the third one bridging Cu2 and Cu3 atoms; Cu5 is bridged in this way to two of its symmetry-related atoms (Cu5^iii^ and Cu5^iv^) [symmetry codes: (iii) 3/2+*x*, 1/2-*y*, -1/2-*z*; (iv) 3/2+*x*, 3/2-*y*, -1/2-*z*] while the third one bridges to the Cu4 atoms. Cu3 and Cu4 atoms show a distorted trigonal planar geometry through coordination to two N atoms from 5-ph-2,2'-bpy and two N/C atoms from two cyanide groups. The Cu3—Cu4 distance is 2.7535 (12) Å, which is not associated with ligand-bridged, hydrogen-bonded, electrostatic-attracted, or /*p*-/*p*-stacked effects, indicating a genuine unsupported Cu^I^—Cu^I^ contact. The unsupported Cu^I^—Cu^I^ contacts are formed between three-coordinate Cu^I^ centers. Furthermore, the ligand-unsupported Cu3—Cu4 distance of 2.7535 (12) Å in (I) is more shorter than those of similar systems containing the unsupported Cu^I^—Cu^I^ interactions (2.9934 (5) Å) (Chen *et al.*, 2009; Zhang *et al.*, 2005), but longer than some other distances found in the literature (2.651 (4) Å) (Zhang *et al.*, 2008).

The adjacent ribbons are packed through intercalation of the lateral 5-ph-2,2'-bpy ligands, in a zipper-like fashion, into two dimensional layers parallel to the *ab* plane (Figure 3).

### Experimental

A mixture of Cu(Ac)_2_ (0.086 g, 0.64 mmol), 5-ph-2,2'-bpy (0.0231 g, 0.1 mmol), K_3_[Fe(CN)_6_] (0.21 g, 0.64 mmol), and water (8 ml) was added to a 15-ml teflon-lined autoclave and heated at 443 K for 3 d. The autoclave was then cooled to room temperature. Orange block crystals of (I) deposited on the wall of container were collected and air-dried.

### Refinement

Hydrogen atoms bound to carbon were placed in calculated positions and refined using a riding model with an isotropic displacement parameter fixed at 1.2 times *U*_eq_ of the atom to which they are attached.

### Crystal data

**Table:**

[Cu_5_(CN)_5_(C_16_H_12_N_2_)_3_]	*F*(000) = 4608
*M**_r_* = 1144.63	*D*_x_ = 1.625 Mg m^−^^3^
Orthorhombic, *P**b**c**a*	Mo *K*α radiation, λ = 0.71073 Å
Hall symbol: -P 2ac 2ab	Cell parameters from 50069 reflections
*a* = 32.132 (8) Å	θ = 1.3–26.2°
*b* = 8.361 (2) Å	µ = 2.29 mm^−^^1^
*c* = 34.836 (9) Å	*T* = 153 K
*V* = 9359 (4) Å^3^	Block, orange
*Z* = 8	0.39 × 0.09 × 0.05 mm

### Data collection

**Table:**

Bruker SMART CCD area-detector diffractometer	9334 independent reflections
Radiation source: fine-focus sealed tube	5271 reflections with *I* > 2σ(*I*)
graphite	*R*_int_ = 0.101
Detector resolution: 9 pixels mm^-1^	θ_max_ = 26.2°, θ_min_ = 1.3°
ω scans	*h* = −39→36
Absorption correction: multi-scan (*SADABS*; Sheldrick, 2004)	*k* = −10→9
*T*_min_ = 0.777, *T*_max_ = 0.884	*l* = −42→36
50069 measured reflections	

### Refinement

**Table:**

Refinement on *F*^2^	Primary atom site location: structure-invariant direct methods
Least-squares matrix: full	Secondary atom site location: difference Fourier map
*R*[*F*^2^ > 2σ(*F*^2^)] = 0.063	Hydrogen site location: inferred from neighbouring sites
*wR*(*F*^2^) = 0.124	H-atom parameters constrained
*S* = 1.05	*w* = 1/[σ^2^(*F*_o_^2^) + (0.0426*P*)^2^ + 0.180*P*] where *P* = (*F*_o_^2^ + 2*F*_c_^2^)/3
9334 reflections	(Δ/σ)_max_ = 0.001
622 parameters	Δρ_max_ = 0.57 e Å^−^^3^
0 restraints	Δρ_min_ = −0.31 e Å^−^^3^

### Special details

**Table:**

Geometry. All e.s.d.'s (except the e.s.d. in the dihedral angle between two l.s. planes) are estimated using the full covariance matrix. The cell e.s.d.'s are taken into account individually in the estimation of e.s.d.'s in distances, angles and torsion angles; correlations between e.s.d.'s in cell parameters are only used when they are defined by crystal symmetry. An approximate (isotropic) treatment of cell e.s.d.'s is used for estimating e.s.d.'s involving l.s. planes.
Refinement. Refinement of *F*^2^ against ALL reflections. The weighted *R*-factor *wR* and goodness of fit *S* are based on *F*^2^, conventional *R*-factors *R* are based on *F*, with *F* set to zero for negative *F*^2^. The threshold expression of *F*^2^ > σ(*F*^2^) is used only for calculating *R*-factors(gt) *etc*. and is not relevant to the choice of reflections for refinement. *R*-factors based on *F*^2^ are statistically about twice as large as those based on *F*, and *R*- factors based on ALL data will be even larger.

### Fractional atomic coordinates and isotropic or equivalent isotropic displacement parameters (Å^2^)

**Table:**

	*x*	*y*	*z*	*U*_iso_*/*U*_eq_	
Cu5	0.292066 (19)	0.21827 (8)	0.14667 (2)	0.0551 (2)	
Cu2	0.575404 (18)	0.29585 (8)	0.114668 (19)	0.0545 (2)	
Cu4	0.42588 (2)	0.44209 (8)	0.10509 (2)	0.0606 (2)	
Cu3	0.433205 (18)	0.12621 (9)	0.083800 (19)	0.0580 (2)	
Cu1	0.655244 (19)	−0.20679 (9)	0.13454 (2)	0.0599 (2)	
N4	0.39861 (11)	−0.0545 (5)	0.10598 (12)	0.0424 (10)	
C16	0.62665 (15)	−0.0124 (6)	0.13124 (14)	0.0449 (13)	
N9	0.34812 (13)	0.2789 (5)	0.13178 (14)	0.0582 (12)	
C24	0.36963 (14)	−0.1127 (6)	0.08183 (15)	0.0459 (13)	
C35	0.26205 (15)	0.0271 (7)	0.15134 (16)	0.0552 (15)	
C42	0.48698 (15)	0.6928 (6)	0.10595 (15)	0.0440 (13)	
N7	0.60739 (13)	0.1016 (6)	0.12532 (13)	0.0593 (13)	
N10	0.25923 (14)	0.4172 (6)	0.15297 (15)	0.0704 (15)	
N2	0.69099 (14)	−0.2899 (5)	0.18068 (14)	0.0601 (12)	
N3	0.40027 (12)	0.0642 (5)	0.03587 (12)	0.0480 (11)	
N8	0.51846 (13)	0.2479 (5)	0.10208 (12)	0.0525 (12)	
N5	0.46821 (11)	0.5935 (5)	0.13071 (12)	0.0436 (10)	
C28	0.39785 (15)	−0.1030 (6)	0.14208 (16)	0.0485 (13)	
H28	0.4184	−0.0640	0.1583	0.058*	
C18	0.48546 (16)	0.2108 (6)	0.09488 (14)	0.0474 (13)	
C25	0.33932 (16)	−0.2137 (7)	0.09461 (18)	0.0622 (16)	
H25	0.3190	−0.2499	0.0777	0.075*	
N1	0.71598 (12)	−0.1934 (5)	0.11268 (13)	0.0498 (11)	
C17	0.60120 (15)	0.4946 (7)	0.11589 (19)	0.0649 (18)	
C45	0.50910 (14)	0.7006 (6)	0.18255 (15)	0.0453 (13)	
C52	0.47889 (15)	0.6003 (6)	0.16736 (16)	0.0492 (14)	
H52	0.4651	0.5325	0.1843	0.059*	
C36	0.37882 (16)	0.3315 (6)	0.12120 (16)	0.0518 (14)	
C23	0.37369 (15)	−0.0561 (6)	0.04180 (15)	0.0485 (13)	
C7	0.79813 (16)	−0.1420 (7)	0.09363 (19)	0.0655 (17)	
H7	0.8260	−0.1262	0.0876	0.079*	
N11	0.62156 (14)	−0.3936 (7)	0.11873 (17)	0.0855 (17)	
C6	0.74613 (16)	−0.2244 (6)	0.13818 (16)	0.0533 (14)	
C44	0.52808 (15)	0.8019 (6)	0.15633 (16)	0.0552 (15)	
H44	0.5484	0.8730	0.1647	0.066*	
C11	0.81251 (16)	0.0388 (7)	0.01947 (19)	0.0633 (16)	
H11	0.8310	0.0635	0.0391	0.076*	
C22	0.78763 (16)	−0.1962 (7)	0.12873 (18)	0.0633 (17)	
H22	0.8083	−0.2150	0.1469	0.076*	
C8	0.76785 (15)	−0.1092 (6)	0.06620 (17)	0.0511 (14)	
C53	0.35257 (17)	−0.1255 (7)	0.01122 (18)	0.0679 (17)	
H53	0.3339	−0.2088	0.0154	0.082*	
C27	0.36864 (16)	−0.2079 (6)	0.15779 (16)	0.0504 (14)	
C46	0.51951 (16)	0.7017 (6)	0.22369 (15)	0.0494 (13)	
C41	0.47349 (15)	0.6814 (6)	0.06576 (15)	0.0459 (13)	
C15	0.75103 (18)	−0.0843 (7)	−0.00283 (19)	0.0686 (17)	
H15	0.7272	−0.1446	0.0018	0.082*	
C9	0.72712 (14)	−0.1379 (6)	0.07856 (17)	0.0537 (15)	
H9	0.7059	−0.1159	0.0612	0.064*	
C5	0.73229 (17)	−0.2867 (7)	0.17556 (17)	0.0587 (15)	
C26	0.33865 (17)	−0.2626 (6)	0.1325 (2)	0.0647 (17)	
H26	0.3181	−0.3323	0.1410	0.078*	
C12	0.82026 (18)	0.0917 (7)	−0.0173 (2)	0.077 (2)	
H12	0.8439	0.1523	−0.0221	0.093*	
N6	0.44479 (13)	0.5695 (5)	0.05824 (13)	0.0543 (12)	
C10	0.77700 (15)	−0.0519 (6)	0.02745 (17)	0.0531 (15)	
C48	0.5685 (2)	0.7490 (7)	0.2747 (2)	0.0748 (19)	
H48	0.5950	0.7791	0.2827	0.090*	
C43	0.51730 (16)	0.7987 (6)	0.11825 (16)	0.0562 (15)	
H43	0.5302	0.8671	0.1009	0.067*	
C47	0.55874 (16)	0.7462 (6)	0.23649 (18)	0.0603 (16)	
H47	0.5789	0.7749	0.2186	0.072*	
C37	0.43204 (17)	0.5525 (8)	0.02189 (19)	0.0722 (18)	
H37	0.4118	0.4760	0.0168	0.087*	
C40	0.48883 (19)	0.7769 (8)	0.03678 (18)	0.0720 (17)	
H40	0.5080	0.8566	0.0425	0.086*	
C19	0.40649 (17)	0.1156 (7)	0.00006 (18)	0.0647 (16)	
H19	0.4255	0.1979	−0.0038	0.078*	
C29	0.36960 (16)	−0.2550 (7)	0.19858 (18)	0.0564 (15)	
C20	0.38641 (19)	0.0541 (8)	−0.03157 (17)	0.0740 (18)	
H20	0.3909	0.0944	−0.0561	0.089*	
C39	0.4762 (2)	0.7557 (8)	−0.00028 (19)	0.080 (2)	
H39	0.4873	0.8184	−0.0198	0.096*	
C2	0.7015 (2)	−0.4057 (9)	0.2428 (2)	0.101 (2)	
H2	0.6900	−0.4434	0.2656	0.122*	
C30	0.35350 (19)	−0.3999 (8)	0.2106 (2)	0.085 (2)	
H30	0.3417	−0.4694	0.1928	0.102*	
C49	0.5390 (2)	0.7073 (7)	0.30098 (19)	0.0761 (19)	
H49	0.5456	0.7082	0.3270	0.091*	
C13	0.7936 (2)	0.0564 (8)	−0.0466 (2)	0.0778 (19)	
H13	0.7993	0.0922	−0.0714	0.093*	
C14	0.75837 (19)	−0.0323 (8)	−0.03953 (19)	0.0748 (18)	
H14	0.7399	−0.0566	−0.0592	0.090*	
C51	0.49049 (19)	0.6607 (7)	0.25143 (18)	0.0681 (17)	
H51	0.4639	0.6300	0.2439	0.082*	
C31	0.3550 (3)	−0.4413 (12)	0.2488 (3)	0.117 (3)	
H31	0.3438	−0.5383	0.2567	0.141*	
C38	0.44699 (19)	0.6412 (9)	−0.00840 (18)	0.0751 (18)	
H38	0.4377	0.6241	−0.0333	0.090*	
C32	0.3725 (3)	−0.3431 (14)	0.2749 (3)	0.112 (4)	
H32	0.3739	−0.3736	0.3006	0.134*	
C1	0.67688 (19)	−0.3510 (7)	0.2134 (2)	0.0776 (19)	
H1	0.6482	−0.3572	0.2166	0.093*	
C34	0.38697 (19)	−0.1556 (8)	0.22580 (19)	0.0785 (19)	
H34	0.3980	−0.0576	0.2184	0.094*	
C3	0.7433 (2)	−0.4026 (10)	0.2372 (2)	0.118 (3)	
H3	0.7613	−0.4401	0.2562	0.142*	
C50	0.5001 (2)	0.6645 (8)	0.2898 (2)	0.080 (2)	
H50	0.4801	0.6378	0.3081	0.096*	
C4	0.75863 (19)	−0.3441 (9)	0.2037 (2)	0.099 (3)	
H4	0.7872	−0.3426	0.1996	0.119*	
C21	0.3595 (2)	−0.0700 (8)	−0.02488 (19)	0.0773 (19)	
H21	0.3457	−0.1171	−0.0455	0.093*	
C33	0.3880 (2)	−0.2005 (11)	0.2638 (2)	0.107 (3)	
H33	0.3996	−0.1320	0.2819	0.128*	

### Atomic displacement parameters (Å^2^)

**Table:**

	*U*^11^	*U*^22^	*U*^33^	*U*^12^	*U*^13^	*U*^23^
Cu5	0.0456 (4)	0.0434 (4)	0.0763 (5)	−0.0033 (3)	0.0000 (3)	0.0026 (4)
Cu2	0.0432 (4)	0.0579 (4)	0.0625 (5)	−0.0053 (3)	−0.0059 (3)	0.0009 (3)
Cu4	0.0566 (4)	0.0606 (5)	0.0647 (5)	−0.0177 (4)	−0.0014 (4)	0.0082 (4)
Cu3	0.0487 (4)	0.0706 (5)	0.0546 (4)	−0.0198 (3)	−0.0097 (3)	0.0056 (4)
Cu1	0.0414 (4)	0.0608 (5)	0.0773 (5)	0.0021 (3)	0.0003 (4)	0.0037 (4)
N4	0.042 (2)	0.040 (3)	0.045 (3)	−0.004 (2)	−0.003 (2)	−0.002 (2)
C16	0.040 (3)	0.046 (3)	0.049 (3)	−0.002 (3)	−0.005 (3)	0.000 (3)
N9	0.045 (3)	0.045 (3)	0.085 (4)	0.003 (2)	0.003 (3)	0.003 (2)
C24	0.038 (3)	0.042 (3)	0.058 (4)	0.005 (2)	−0.004 (3)	−0.011 (3)
C35	0.039 (3)	0.043 (4)	0.084 (4)	0.004 (3)	0.000 (3)	0.002 (3)
C42	0.042 (3)	0.037 (3)	0.052 (4)	0.000 (2)	0.013 (3)	−0.002 (3)
N7	0.057 (3)	0.059 (3)	0.062 (3)	−0.013 (3)	−0.010 (2)	0.000 (3)
N10	0.052 (3)	0.042 (3)	0.117 (5)	−0.003 (2)	0.003 (3)	−0.002 (3)
N2	0.054 (3)	0.057 (3)	0.070 (4)	0.005 (2)	0.010 (3)	0.007 (3)
N3	0.041 (2)	0.060 (3)	0.043 (3)	−0.002 (2)	−0.004 (2)	−0.003 (2)
N8	0.042 (3)	0.054 (3)	0.061 (3)	−0.007 (2)	−0.010 (2)	0.004 (2)
N5	0.044 (2)	0.042 (3)	0.044 (3)	−0.007 (2)	0.005 (2)	0.004 (2)
C28	0.050 (3)	0.044 (3)	0.052 (4)	−0.002 (3)	−0.004 (3)	−0.006 (3)
C18	0.049 (3)	0.055 (3)	0.039 (3)	−0.004 (3)	0.000 (3)	0.003 (3)
C25	0.046 (3)	0.063 (4)	0.078 (5)	−0.017 (3)	−0.009 (3)	−0.008 (3)
N1	0.040 (2)	0.051 (3)	0.058 (3)	−0.001 (2)	−0.003 (2)	−0.003 (2)
C17	0.034 (3)	0.043 (4)	0.117 (6)	−0.009 (3)	−0.001 (3)	−0.009 (3)
C45	0.040 (3)	0.041 (3)	0.055 (4)	−0.001 (3)	0.004 (3)	−0.003 (3)
C52	0.052 (3)	0.043 (3)	0.053 (4)	−0.001 (3)	0.007 (3)	0.010 (3)
C36	0.044 (3)	0.050 (4)	0.062 (4)	0.003 (3)	−0.007 (3)	0.006 (3)
C23	0.045 (3)	0.052 (4)	0.048 (4)	0.004 (3)	−0.009 (3)	−0.007 (3)
C7	0.032 (3)	0.083 (5)	0.082 (5)	−0.009 (3)	0.003 (3)	−0.016 (4)
N11	0.052 (3)	0.064 (4)	0.140 (5)	0.016 (3)	0.007 (3)	−0.011 (4)
C6	0.044 (3)	0.056 (4)	0.060 (4)	0.000 (3)	−0.004 (3)	−0.011 (3)
C44	0.048 (3)	0.054 (4)	0.064 (4)	−0.014 (3)	0.007 (3)	−0.004 (3)
C11	0.051 (3)	0.057 (4)	0.082 (5)	−0.003 (3)	0.016 (3)	−0.009 (3)
C22	0.039 (3)	0.084 (5)	0.066 (4)	−0.004 (3)	−0.008 (3)	−0.003 (4)
C8	0.037 (3)	0.047 (3)	0.069 (4)	−0.005 (3)	0.006 (3)	−0.012 (3)
C53	0.069 (4)	0.063 (4)	0.071 (5)	−0.007 (3)	−0.018 (4)	−0.010 (4)
C27	0.044 (3)	0.042 (3)	0.066 (4)	0.004 (3)	0.010 (3)	0.000 (3)
C46	0.056 (3)	0.042 (3)	0.050 (4)	−0.001 (3)	0.002 (3)	−0.006 (3)
C41	0.042 (3)	0.048 (4)	0.047 (4)	0.004 (3)	0.012 (3)	0.006 (3)
C15	0.060 (4)	0.075 (5)	0.071 (5)	−0.014 (3)	0.017 (4)	−0.010 (4)
C9	0.033 (3)	0.056 (4)	0.072 (4)	0.001 (3)	−0.009 (3)	−0.006 (3)
C5	0.050 (3)	0.060 (4)	0.067 (4)	0.002 (3)	−0.011 (3)	−0.006 (3)
C26	0.057 (4)	0.046 (4)	0.091 (5)	−0.014 (3)	0.007 (4)	0.005 (3)
C12	0.056 (4)	0.058 (4)	0.117 (6)	−0.002 (3)	0.029 (4)	−0.004 (4)
N6	0.051 (3)	0.059 (3)	0.053 (3)	−0.003 (2)	−0.009 (2)	0.006 (2)
C10	0.039 (3)	0.051 (4)	0.069 (4)	−0.002 (3)	0.013 (3)	−0.012 (3)
C48	0.075 (4)	0.076 (5)	0.073 (5)	0.005 (4)	−0.018 (4)	−0.006 (4)
C43	0.063 (4)	0.052 (4)	0.053 (4)	−0.013 (3)	0.010 (3)	0.007 (3)
C47	0.048 (3)	0.068 (4)	0.066 (4)	0.003 (3)	0.002 (3)	−0.001 (3)
C37	0.067 (4)	0.082 (5)	0.067 (5)	−0.009 (3)	−0.011 (4)	0.006 (4)
C40	0.082 (4)	0.079 (5)	0.055 (4)	−0.015 (4)	0.016 (4)	0.000 (4)
C19	0.056 (3)	0.082 (5)	0.057 (4)	0.001 (3)	−0.004 (3)	−0.002 (4)
C29	0.056 (3)	0.056 (4)	0.057 (4)	0.012 (3)	0.014 (3)	0.010 (3)
C20	0.086 (5)	0.089 (5)	0.047 (4)	0.017 (4)	−0.007 (4)	−0.007 (4)
C39	0.093 (5)	0.088 (6)	0.059 (5)	−0.001 (4)	0.018 (4)	0.016 (4)
C2	0.093 (5)	0.130 (7)	0.081 (6)	0.004 (5)	−0.004 (5)	0.031 (5)
C30	0.088 (5)	0.055 (5)	0.112 (6)	0.003 (4)	0.040 (4)	0.022 (4)
C49	0.116 (6)	0.056 (4)	0.056 (4)	0.011 (4)	−0.016 (5)	−0.006 (3)
C13	0.078 (5)	0.071 (5)	0.085 (5)	0.005 (4)	0.035 (4)	0.002 (4)
C14	0.075 (4)	0.082 (5)	0.067 (5)	−0.006 (4)	0.012 (4)	−0.011 (4)
C51	0.077 (4)	0.070 (5)	0.057 (4)	−0.018 (3)	−0.001 (4)	−0.001 (3)
C31	0.102 (7)	0.111 (8)	0.139 (9)	0.032 (6)	0.053 (6)	0.067 (7)
C38	0.081 (5)	0.097 (5)	0.047 (4)	0.001 (4)	−0.005 (4)	0.005 (4)
C32	0.091 (7)	0.165 (11)	0.079 (7)	0.047 (6)	0.032 (5)	0.056 (7)
C1	0.066 (4)	0.077 (5)	0.090 (5)	0.007 (3)	0.005 (4)	0.022 (4)
C34	0.092 (5)	0.088 (5)	0.055 (4)	0.006 (4)	0.011 (4)	0.010 (4)
C3	0.085 (6)	0.166 (9)	0.103 (7)	−0.007 (6)	−0.030 (5)	0.066 (6)
C50	0.098 (5)	0.081 (5)	0.060 (5)	−0.013 (4)	0.009 (4)	0.004 (4)
C4	0.057 (4)	0.144 (7)	0.097 (6)	−0.013 (4)	−0.021 (4)	0.040 (5)
C21	0.088 (5)	0.081 (5)	0.063 (5)	0.006 (4)	−0.030 (4)	−0.020 (4)
C33	0.108 (6)	0.146 (9)	0.067 (6)	0.022 (6)	0.013 (5)	0.003 (5)

### Geometric parameters (Å, °)

**Table:**

Cu1—C16	1.871 (5)	C53—C21	1.359 (8)
Cu1—N11	1.978 (6)	C53—H53	0.9300
Cu1—N2	2.095 (5)	C27—C26	1.384 (7)
Cu1—N1	2.098 (4)	C27—C29	1.475 (7)
Cu2—C17	1.857 (6)	C46—C51	1.386 (7)
Cu2—N8	1.924 (4)	C46—C47	1.388 (7)
Cu2—N7	1.958 (5)	C41—N6	1.340 (6)
Cu3—C18	1.862 (5)	C41—C40	1.378 (7)
Cu3—N4	2.029 (4)	C15—C14	1.371 (8)
Cu3—N3	2.044 (4)	C15—C10	1.372 (7)
Cu3—Cu4	2.7535 (12)	C15—H15	0.9300
Cu4—C36	1.859 (6)	C9—H9	0.9300
Cu4—N6	2.041 (4)	C5—C4	1.381 (8)
Cu4—N5	2.062 (4)	C26—H26	0.9300
Cu5—C35	1.874 (6)	C12—C13	1.367 (8)
Cu5—N9	1.942 (4)	C12—H12	0.9300
Cu5—N10	1.982 (5)	N6—C37	1.338 (6)
N4—C28	1.321 (6)	C48—C49	1.363 (8)
N4—C24	1.346 (6)	C48—C47	1.368 (8)
C16—N7	1.155 (6)	C48—H48	0.9300
N9—C36	1.141 (6)	C43—H43	0.9300
C24—C25	1.364 (7)	C47—H47	0.9300
C24—C23	1.478 (7)	C37—C38	1.376 (8)
C35—N10^i^	1.147 (6)	C37—H37	0.9300
C42—N5	1.340 (6)	C40—C39	1.365 (8)
C42—C43	1.385 (7)	C40—H40	0.9300
C42—C41	1.469 (7)	C19—C20	1.377 (7)
N10—C35^ii^	1.147 (6)	C19—H19	0.9300
N2—C1	1.328 (7)	C29—C34	1.379 (8)
N2—C5	1.339 (6)	C29—C30	1.382 (7)
N3—C19	1.335 (6)	C20—C21	1.370 (8)
N3—C23	1.335 (6)	C20—H20	0.9300
N8—C18	1.133 (5)	C39—C38	1.370 (8)
N5—C52	1.323 (6)	C39—H39	0.9300
C28—C27	1.396 (7)	C2—C3	1.358 (8)
C28—H28	0.9300	C2—C1	1.373 (8)
C25—C26	1.382 (7)	C2—H2	0.9300
C25—H25	0.9300	C30—C31	1.378 (10)
N1—C9	1.326 (6)	C30—H30	0.9300
N1—C6	1.340 (6)	C49—C50	1.356 (8)
C17—N11^iii^	1.146 (6)	C49—H49	0.9300
C45—C44	1.387 (7)	C13—C14	1.376 (8)
C45—C52	1.388 (6)	C13—H13	0.9300
C45—C46	1.472 (7)	C14—H14	0.9300
C52—H52	0.9300	C51—C50	1.374 (8)
C23—C53	1.390 (7)	C51—H51	0.9300
C7—C22	1.347 (7)	C31—C32	1.349 (11)
C7—C8	1.391 (7)	C31—H31	0.9300
C7—H7	0.9300	C38—H38	0.9300
N11—C17^iv^	1.146 (6)	C32—C33	1.350 (11)
C6—C22	1.393 (7)	C32—H32	0.9300
C6—C5	1.471 (7)	C1—H1	0.9300
C44—C43	1.371 (7)	C34—C33	1.375 (9)
C44—H44	0.9300	C34—H34	0.9300
C11—C12	1.376 (8)	C3—C4	1.360 (9)
C11—C10	1.398 (7)	C3—H3	0.9300
C11—H11	0.9300	C50—H50	0.9300
C22—H22	0.9300	C4—H4	0.9300
C8—C9	1.398 (6)	C21—H21	0.9300
C8—C10	1.463 (7)	C33—H33	0.9300
			
C35—Cu5—N9	136.3 (2)	N6—C41—C42	115.8 (5)
C35—Cu5—N10	115.6 (2)	C40—C41—C42	123.7 (5)
N9—Cu5—N10	107.72 (18)	C14—C15—C10	123.4 (6)
C17—Cu2—N8	128.0 (2)	C14—C15—H15	118.3
C17—Cu2—N7	120.2 (2)	C10—C15—H15	118.3
N8—Cu2—N7	111.70 (18)	N1—C9—C8	126.1 (5)
C36—Cu4—N6	138.0 (2)	N1—C9—H9	117.0
C36—Cu4—N5	135.3 (2)	C8—C9—H9	117.0
N6—Cu4—N5	80.17 (17)	N2—C5—C4	120.4 (6)
C36—Cu4—Cu3	70.95 (17)	N2—C5—C6	115.1 (5)
N6—Cu4—Cu3	105.05 (13)	C4—C5—C6	124.4 (6)
N5—Cu4—Cu3	130.49 (11)	C25—C26—C27	120.0 (5)
C18—Cu3—N4	134.27 (19)	C25—C26—H26	120.0
C18—Cu3—N3	137.10 (19)	C27—C26—H26	120.0
N4—Cu3—N3	80.70 (17)	C13—C12—C11	120.9 (6)
C18—Cu3—Cu4	69.94 (16)	C13—C12—H12	119.6
N4—Cu3—Cu4	124.40 (11)	C11—C12—H12	119.6
N3—Cu3—Cu4	114.78 (12)	C37—N6—C41	118.0 (5)
C16—Cu1—N11	113.6 (2)	C37—N6—Cu4	127.6 (4)
C16—Cu1—N2	127.2 (2)	C41—N6—Cu4	114.4 (4)
N11—Cu1—N2	104.6 (2)	C15—C10—C11	116.8 (6)
C16—Cu1—N1	112.85 (19)	C15—C10—C8	121.5 (5)
N11—Cu1—N1	116.74 (18)	C11—C10—C8	121.7 (5)
N2—Cu1—N1	77.64 (18)	C49—C48—C47	119.3 (6)
C28—N4—C24	118.1 (4)	C49—C48—H48	120.3
C28—N4—Cu3	126.9 (3)	C47—C48—H48	120.3
C24—N4—Cu3	114.2 (3)	C44—C43—C42	119.3 (5)
N7—C16—Cu1	172.4 (5)	C44—C43—H43	120.4
C36—N9—Cu5	171.4 (4)	C42—C43—H43	120.4
N4—C24—C25	120.9 (5)	C48—C47—C46	121.7 (6)
N4—C24—C23	114.4 (4)	C48—C47—H47	119.2
C25—C24—C23	124.7 (5)	C46—C47—H47	119.2
N10^i^—C35—Cu5	174.1 (5)	N6—C37—C38	124.2 (6)
N5—C42—C43	120.9 (5)	N6—C37—H37	117.9
N5—C42—C41	116.2 (4)	C38—C37—H37	117.9
C43—C42—C41	122.9 (5)	C39—C40—C41	120.8 (6)
C16—N7—Cu2	179.1 (5)	C39—C40—H40	119.6
C35^ii^—N10—Cu5	169.9 (5)	C41—C40—H40	119.6
C1—N2—C5	117.4 (5)	N3—C19—C20	123.9 (6)
C1—N2—Cu1	126.8 (4)	N3—C19—H19	118.1
C5—N2—Cu1	115.7 (4)	C20—C19—H19	118.1
C19—N3—C23	118.9 (5)	C34—C29—C30	118.2 (6)
C19—N3—Cu3	127.2 (4)	C34—C29—C27	120.7 (6)
C23—N3—Cu3	113.3 (4)	C30—C29—C27	121.1 (6)
C18—N8—Cu2	176.1 (5)	C21—C20—C19	116.3 (6)
C52—N5—C42	118.6 (4)	C21—C20—H20	121.9
C52—N5—Cu4	128.0 (3)	C19—C20—H20	121.9
C42—N5—Cu4	113.5 (3)	C40—C39—C38	119.3 (6)
N4—C28—C27	125.3 (5)	C40—C39—H39	120.3
N4—C28—H28	117.3	C38—C39—H39	120.3
C27—C28—H28	117.3	C3—C2—C1	117.2 (7)
N8—C18—Cu3	173.6 (5)	C3—C2—H2	121.4
C24—C25—C26	120.4 (5)	C1—C2—H2	121.4
C24—C25—H25	119.8	C31—C30—C29	119.9 (8)
C26—C25—H25	119.8	C31—C30—H30	120.0
C9—N1—C6	117.8 (4)	C29—C30—H30	120.0
C9—N1—Cu1	126.5 (3)	C50—C49—C48	121.1 (6)
C6—N1—Cu1	115.0 (4)	C50—C49—H49	119.5
N11^iii^—C17—Cu2	170.8 (5)	C48—C49—H49	119.5
C44—C45—C52	115.2 (5)	C12—C13—C14	119.8 (6)
C44—C45—C46	122.5 (5)	C12—C13—H13	120.1
C52—C45—C46	122.3 (5)	C14—C13—H13	120.1
N5—C52—C45	125.1 (5)	C15—C14—C13	118.7 (6)
N5—C52—H52	117.4	C15—C14—H14	120.6
C45—C52—H52	117.4	C13—C14—H14	120.6
N9—C36—Cu4	172.8 (5)	C50—C51—C46	121.5 (6)
N3—C23—C53	120.6 (5)	C50—C51—H51	119.3
N3—C23—C24	116.3 (5)	C46—C51—H51	119.3
C53—C23—C24	123.1 (5)	C32—C31—C30	121.0 (9)
C22—C7—C8	121.0 (5)	C32—C31—H31	119.5
C22—C7—H7	119.5	C30—C31—H31	119.5
C8—C7—H7	119.5	C39—C38—C37	117.2 (6)
C17^iv^—N11—Cu1	168.8 (6)	C39—C38—H38	121.4
N1—C6—C22	120.2 (5)	C37—C38—H38	121.4
N1—C6—C5	115.9 (5)	C31—C32—C33	119.8 (9)
C22—C6—C5	123.9 (5)	C31—C32—H32	120.1
C43—C44—C45	120.9 (5)	C33—C32—H32	120.1
C43—C44—H44	119.5	N2—C1—C2	124.8 (6)
C45—C44—H44	119.5	N2—C1—H1	117.6
C12—C11—C10	120.5 (6)	C2—C1—H1	117.6
C12—C11—H11	119.8	C33—C34—C29	120.5 (7)
C10—C11—H11	119.8	C33—C34—H34	119.8
C7—C22—C6	120.7 (5)	C29—C34—H34	119.8
C7—C22—H22	119.6	C2—C3—C4	119.1 (7)
C6—C22—H22	119.6	C2—C3—H3	120.4
C7—C8—C9	114.2 (5)	C4—C3—H3	120.4
C7—C8—C10	123.9 (5)	C49—C50—C51	119.5 (6)
C9—C8—C10	121.9 (5)	C49—C50—H50	120.3
C21—C53—C23	119.1 (6)	C51—C50—H50	120.3
C21—C53—H53	120.4	C3—C4—C5	120.9 (6)
C23—C53—H53	120.4	C3—C4—H4	119.6
C26—C27—C28	115.2 (5)	C5—C4—H4	119.6
C26—C27—C29	122.7 (5)	C53—C21—C20	121.3 (6)
C28—C27—C29	122.1 (5)	C53—C21—H21	119.4
C51—C46—C47	117.0 (5)	C20—C21—H21	119.4
C51—C46—C45	121.6 (5)	C32—C33—C34	120.6 (9)
C47—C46—C45	121.4 (5)	C32—C33—H33	119.7
N6—C41—C40	120.5 (5)	C34—C33—H33	119.7
			
C36—Cu4—Cu3—C18	−147.4 (2)	C44—C45—C46—C47	−28.7 (8)
N6—Cu4—Cu3—C18	76.5 (2)	C52—C45—C46—C47	152.8 (5)
N5—Cu4—Cu3—C18	−13.3 (2)	N5—C42—C41—N6	−2.0 (6)
C36—Cu4—Cu3—N4	−16.8 (2)	C43—C42—C41—N6	178.0 (5)
N6—Cu4—Cu3—N4	−152.93 (18)	N5—C42—C41—C40	178.5 (5)
N5—Cu4—Cu3—N4	117.2 (2)	C43—C42—C41—C40	−1.6 (8)
C36—Cu4—Cu3—N3	79.0 (2)	C6—N1—C9—C8	0.6 (8)
N6—Cu4—Cu3—N3	−57.16 (18)	Cu1—N1—C9—C8	170.7 (4)
N5—Cu4—Cu3—N3	−147.0 (2)	C7—C8—C9—N1	−1.0 (8)
C18—Cu3—N4—C28	32.4 (5)	C10—C8—C9—N1	178.2 (5)
N3—Cu3—N4—C28	−176.4 (4)	C1—N2—C5—C4	−0.5 (9)
Cu4—Cu3—N4—C28	−62.6 (4)	Cu1—N2—C5—C4	−177.7 (5)
C18—Cu3—N4—C24	−157.7 (3)	C1—N2—C5—C6	177.4 (5)
N3—Cu3—N4—C24	−6.4 (3)	Cu1—N2—C5—C6	0.2 (6)
Cu4—Cu3—N4—C24	107.3 (3)	N1—C6—C5—N2	−6.2 (7)
C28—N4—C24—C25	2.6 (7)	C22—C6—C5—N2	173.3 (5)
Cu3—N4—C24—C25	−168.3 (4)	N1—C6—C5—C4	171.6 (6)
C28—N4—C24—C23	−178.1 (4)	C22—C6—C5—C4	−8.9 (9)
Cu3—N4—C24—C23	11.0 (5)	C24—C25—C26—C27	0.6 (9)
C35—Cu5—N10—C35^ii^	−74 (3)	C28—C27—C26—C25	0.4 (8)
N9—Cu5—N10—C35^ii^	101 (3)	C29—C27—C26—C25	179.8 (5)
C16—Cu1—N2—C1	77.1 (6)	C10—C11—C12—C13	−0.3 (9)
N11—Cu1—N2—C1	−58.7 (5)	C40—C41—N6—C37	0.8 (8)
N1—Cu1—N2—C1	−173.5 (5)	C42—C41—N6—C37	−178.8 (5)
C16—Cu1—N2—C5	−106.0 (4)	C40—C41—N6—Cu4	−179.6 (4)
N11—Cu1—N2—C5	118.2 (4)	C42—C41—N6—Cu4	0.8 (5)
N1—Cu1—N2—C5	3.4 (4)	C36—Cu4—N6—C37	−27.8 (6)
C18—Cu3—N3—C19	−21.0 (6)	N5—Cu4—N6—C37	179.8 (5)
N4—Cu3—N3—C19	−170.6 (5)	Cu3—Cu4—N6—C37	50.3 (5)
Cu4—Cu3—N3—C19	65.7 (5)	C36—Cu4—N6—C41	152.6 (4)
C18—Cu3—N3—C23	149.8 (4)	N5—Cu4—N6—C41	0.2 (3)
N4—Cu3—N3—C23	0.2 (3)	Cu3—Cu4—N6—C41	−129.3 (3)
Cu4—Cu3—N3—C23	−123.5 (3)	C14—C15—C10—C11	−0.1 (9)
C43—C42—N5—C52	0.8 (7)	C14—C15—C10—C8	179.7 (5)
C41—C42—N5—C52	−179.2 (4)	C12—C11—C10—C15	0.1 (8)
C43—C42—N5—Cu4	−177.9 (4)	C12—C11—C10—C8	−179.7 (5)
C41—C42—N5—Cu4	2.1 (5)	C7—C8—C10—C15	151.7 (5)
C36—Cu4—N5—C52	26.3 (5)	C9—C8—C10—C15	−27.4 (8)
N6—Cu4—N5—C52	−179.8 (4)	C7—C8—C10—C11	−28.5 (8)
Cu3—Cu4—N5—C52	−78.4 (4)	C9—C8—C10—C11	152.4 (5)
C36—Cu4—N5—C42	−155.1 (3)	C45—C44—C43—C42	0.1 (8)
N6—Cu4—N5—C42	−1.3 (3)	N5—C42—C43—C44	−0.1 (8)
Cu3—Cu4—N5—C42	100.2 (3)	C41—C42—C43—C44	179.9 (5)
C24—N4—C28—C27	−1.6 (7)	C49—C48—C47—C46	−0.1 (9)
Cu3—N4—C28—C27	168.0 (4)	C51—C46—C47—C48	0.2 (8)
N4—C24—C25—C26	−2.2 (8)	C45—C46—C47—C48	179.3 (5)
C23—C24—C25—C26	178.6 (5)	C41—N6—C37—C38	1.1 (9)
C16—Cu1—N1—C9	−51.8 (5)	Cu4—N6—C37—C38	−178.5 (5)
N11—Cu1—N1—C9	82.6 (5)	N6—C41—C40—C39	−2.3 (9)
N2—Cu1—N1—C9	−177.2 (5)	C42—C41—C40—C39	177.3 (5)
C16—Cu1—N1—C6	118.6 (4)	C23—N3—C19—C20	1.3 (8)
N11—Cu1—N1—C6	−107.1 (4)	Cu3—N3—C19—C20	171.7 (4)
N2—Cu1—N1—C6	−6.8 (4)	C26—C27—C29—C34	−152.8 (6)
C42—N5—C52—C45	−1.6 (7)	C28—C27—C29—C34	26.5 (8)
Cu4—N5—C52—C45	176.9 (4)	C26—C27—C29—C30	27.7 (8)
C44—C45—C52—N5	1.5 (7)	C28—C27—C29—C30	−153.0 (5)
C46—C45—C52—N5	−179.9 (5)	N3—C19—C20—C21	−1.8 (9)
C19—N3—C23—C53	−0.6 (7)	C41—C40—C39—C38	2.0 (10)
Cu3—N3—C23—C53	−172.3 (4)	C34—C29—C30—C31	0.2 (9)
C19—N3—C23—C24	177.2 (4)	C27—C29—C30—C31	179.7 (6)
Cu3—N3—C23—C24	5.6 (5)	C47—C48—C49—C50	−0.6 (9)
N4—C24—C23—N3	−11.1 (6)	C11—C12—C13—C14	0.4 (9)
C25—C24—C23—N3	168.1 (5)	C10—C15—C14—C13	0.3 (9)
N4—C24—C23—C53	166.6 (5)	C12—C13—C14—C15	−0.4 (9)
C25—C24—C23—C53	−14.1 (8)	C47—C46—C51—C50	0.2 (8)
C16—Cu1—N11—C17^iv^	−100 (2)	C45—C46—C51—C50	−178.9 (5)
N2—Cu1—N11—C17^iv^	43 (2)	C29—C30—C31—C32	−0.9 (12)
N1—Cu1—N11—C17^iv^	126 (2)	C40—C39—C38—C37	−0.2 (10)
C9—N1—C6—C22	0.8 (8)	N6—C37—C38—C39	−1.3 (10)
Cu1—N1—C6—C22	−170.4 (4)	C30—C31—C32—C33	1.5 (14)
C9—N1—C6—C5	−179.7 (5)	C5—N2—C1—C2	2.4 (10)
Cu1—N1—C6—C5	9.1 (6)	Cu1—N2—C1—C2	179.3 (5)
C52—C45—C44—C43	−0.6 (7)	C3—C2—C1—N2	−2.7 (12)
C46—C45—C44—C43	−179.3 (5)	C30—C29—C34—C33	−0.1 (9)
C8—C7—C22—C6	1.5 (9)	C27—C29—C34—C33	−179.6 (5)
N1—C6—C22—C7	−1.9 (8)	C1—C2—C3—C4	1.1 (13)
C5—C6—C22—C7	178.7 (6)	C48—C49—C50—C51	1.0 (10)
C22—C7—C8—C9	−0.1 (8)	C46—C51—C50—C49	−0.8 (9)
C22—C7—C8—C10	−179.3 (5)	C2—C3—C4—C5	0.7 (13)
N3—C23—C53—C21	0.4 (8)	N2—C5—C4—C3	−1.0 (11)
C24—C23—C53—C21	−177.3 (5)	C6—C5—C4—C3	−178.7 (7)
N4—C28—C27—C26	0.0 (8)	C23—C53—C21—C20	−0.9 (9)
N4—C28—C27—C29	−179.3 (5)	C19—C20—C21—C53	1.5 (9)
C44—C45—C46—C51	150.4 (5)	C31—C32—C33—C34	−1.4 (13)
C52—C45—C46—C51	−28.2 (8)	C29—C34—C33—C32	0.7 (11)

Symmetry codes: (i) −*x*+1/2, *y*−1/2, *z*; (ii) −*x*+1/2, *y*+1/2, *z*; (iii) *x*, *y*+1, *z*; (iv) *x*, *y*−1, *z*.
